# Obstetric fistula management and predictors of successful closure among women attending a public tertiary hospital in Rwanda: a retrospective review of records

**DOI:** 10.1186/s13104-015-1771-y

**Published:** 2015-12-12

**Authors:** Tekle G. Egziabher, Ngoga Eugene, Karenzi Ben, Kateera Fredrick

**Affiliations:** Rwanda Military Hospital, P.O. BOX 3377, Kigali, Rwanda

**Keywords:** Obstetric, Fistula, Prolonged labour, Repair outcomes, Rwanda

## Abstract

**Background:**

Globally, 50,000–100,000 women develop obstetric fistula annually. At least 33,000 of these women live in Sub-Saharan Africa where limitations in quality obstetric care and fistula corrective repairs are prevalent. Among women with fistula seeking care at public health facilities in resource-limited settings, there is paucity of data on quality of care received. The aim of this study was to characterize obstetric fistula among Rwandan women managed at a public tertiary hospital and evaluate for predictors of successful fistula closures.

**Methods:**

A retrospective review of records for all obstetric fistula women managed at a public referral health facility between 2007 and 2013 was performed. Patient socio-demographics, obstetric characteristics and fistula repair outcomes data were reviewed. A multivariate logistic regression model was used to analyse for predictors of successful fistula repair outcomes.

**Results:**

A total of 272 women aged between 16 to 78 years and with a mean age of 34.6 years were included. Of these, 93 (34.2 %), 48 (17.6 %), 65 (24 %) and 64 (23 %) women had vesico-vaginal fistula, recto-vaginal fistula, urethro-vaginal fistula and vesico-uteral fistula types, respectively. Successful fistula closure was achieved among 86.3 %. Women with fistula who reported being in labour for ≥3 days, having ≥1 previous fistula repair attempt, and having lived with the fistula for >1 year, had significantly lower odds of successful repair outcomes.

**Conclusions:**

Among 272 women with obstetric fistula managed in this study, 69.5 and 26.5 % of their fistula were causally associated with obstructed labour complications and iatrogenic factors, respectively. Successful fistula closure rates of about 89 % among women of index repair attempt were achieved. Conversely, reported histories of ≥3 days in labour, ≥1 previous failed attempts at repair and a fistula duration of >1 year, were significant determinants of failed fistula closures. To effectively mitigate obstetric fistula burden in Rwanda, a comprehensive package of services including quality emergency obstetric care, increased availability of and access to quality fistula repair, active surveillance to identify community-based women with fistula and a strong political will towards effective fistula care, are recommended.

## Background

Obstructed labour affects more than 6 million women annually with over 90 % of these women living in the world’s poorest areas with limited access to emergency obstetric care and quality fistula corrective services [1, 2]. Over 40,000 women are estimated to die from neglected obstructed labour annually [3].

An obstetric fistula is a hole between the vagina and bladder (vesicovaginal fistula) or the vagina and rectum (rectovaginal fistula) that is caused by prolonged obstructed labour, leaving a woman incontinent of urine or faeces, respectively [4]. In the developed world, obstetric fistula cases are rare with the commonest aetiologies of the few cases observed including malignancies, radiation therapy, pelvic surgery, infections and trauma. In the developing world where fistula are prevalent, most fistula arise from tissue destruction associated with prolonged pressure of the foetal head or presenting parts during obstructed labour.

Conservatively, an estimated 2–3 million women live with untreated obstetric fistula. The World Health Organization (WHO) estimates that about 50,000–100, 000 women develop obstetric fistula annually with at least 33,000 of these located in sub-Saharan Africa (SSA) [5–7]. Unfortunately, data on the burden of obstetric fistula are scarce with the available information generally drawn from self-reports, personal communications from surgical teams, data aggregated from advocacy groups and organizations like Engender Health and United Nations Population Fund (UNFPA) and record reviews of hospital services. The estimates generated from these are often incomplete and mostly inaccurate and hence, the reported statistics are generally considered underestimates of the fistula burden. In a recent review that aimed at estimating the global prevalence and incidence of obstetric fistula, only 19 studies were selected: Most of these studies did not have a nationally representative sample and very few had been conducted in South Asia. This review estimated the total number of fistula and number of new fistula cases in the two most affected regions of SSA and South Asia to be just over one million and an estimated 6000 cases per year, respectively [8].

In developing counties, obstetric fistula are a public health challenge associated with inadequate emergency obstetric care, limited fistula repair services and a lack of fistula surgeons to manage the affected women [9]. Compounding this challenge is the limited knowledge among those affected on where to access fistula care. In the East African region for example, at least 80 % of women with fistula are estimated to be lacking fistula repair services each year [10]. In addition, fistula affect mainly marginalized groups of uneducated young females mostly living in geographical remote settings with limited to no obstetric care services and in countries where fistula care in not prioritization. These factors collectively serve to keep the fistula problem poorly characterized and a relative hidden public health challenge from policy markers and programme planners, and at all levels of health systems. Some of fistula associated complications include urinary, faecal or combined incontinence [11], physical and psychosocial morbidity including societal stigmatization [12, 13], marital breakdowns including divorces and separation [14, 15], loss of income due to difficulty in securing a job [16], and reproductive system complications like infertility and amenorrhea [17, 18].

Currently in Rwanda, there are no available accurate data on the prevalence or incidence of obstetric fistula. Anecdotal information from health facilities and occasional surgical camps targeting management of community based obstetric fistula cases suggest that fistula is a public health concern. By end of 2014, on-going fistula repair programs were available primariry at two referrals [Ruhengeri and Rwanda Military Hospitals (RMH)] and the University Teaching Hospital of Kigali. According to the fistula care project-Rwanda, 1190 fistula cases were reported to have been repaired between 2006 and 2011 [19]. Since June 2012, the Ministry of Health—Rwanda, in collaboration with implementing partners, as part of reinforcing health facility capacity to provide care and treatment to mothers with fistula, set up a target of establishing a 3 level hierarchical fistula repair model including, (1) two level-3 facilities with advanced fistula surgeons mandated with offering fistula repair services and training other medical personnel in fistula care management, (2) three level-2 facilities with capacity to provide simple fistula repair on a routine basis and to refer complex cases to level-3 facilities, and (3) three level-1 facilities with equipment to support fistula prevention and/or act as outreach sites for community fistula repair projects [19]. Following this increased attention to the fistula issue, the paucity of local data must be addressed and  thus, this study aimed at characterizing obstetric fistula among Rwandan women managed at a public tertiary hospital and evaluate for predictors of successful fistula closures.

## Methods

### Study design and period

A retrospective review of medical records for women with obstetric fistula managed between January 2007 and December 2013 was conducted.

### Study site

Rwanda Military Hospital (RMH) is a tertiary training and referral hospital located in Kigali city and serving the general population. Within the study period, RMH employed a full time experienced fistula surgeon who managed all study participants included in this analysis.

### Inclusion and exclusion criteria

All (272) women who visited RMH either as referrals or self-initiated and who were diagnosed with and managed for obstetric fistula were included in this study. In contrast, all women whose fistula repairs were conducted elsewhere but who reported for outpatient evaluation only or those whose repairs were done at RMH but outside the stated study period, and those who did not return for post-operative review 6–8 weeks after surgical management, were excluded from the analysis.

### Data management and variable definitions

Data was manually extracted from patient’s files and entered into an excel sheet by two independent data collectors to ensure accuracy. Data on (1) socio-demographics including age at time of clinical evaluation, area of residence and marital status (married, living together, single, divorced and widowed), (2) obstetric history including parity, mode of delivery for the pregnancy associated with the fistula (vaginal vs. caesarean section), duration (in days) of labour prior to fistula development, associated cause of fistula (abortion, infection, caesarean section, other surgery, obstructed labour and perineal tear), duration with fistula (in months), and fistula repair experience including type of access for fistula repair (virginal vs. abdominal), repair outcome (successful vs. failed) and number of repair attempts done, was aggregated. At 6–8 weeks after surgical management, a follow-up visit was conducted where repair outcome was assigned. A failed repair was defined as presence of continued leakage at the 6–8 weeks post repart follow up visit while a successful repair was defined as fistula closure with no leakage (with or with out stress incontinence) at the follow-up visit. Assessment of outcomes was based on the Waaldijik’s classification—a classification that is useful in predicting outcome and planning treatment [20]. In this study, fistula types were assigned by their primary anatomical pathology as (1) VVF, (2) RVF, (3) uretero-vaginal fistula (UVF), (4) vesico-uterine fistulas (VUF), and (5) uro-rectal fistulae (URF).

### Study participant follow-up visits

All cases in this study were managed and evaluated by the same obstetric surgeon. In summary, study subjects were admitted for evaluation, corrective surgery and a 14–21 days post-repair observational and healing period with the more complicated cases staying for up to a 21-day period. Upon discharge, participants were scheduled for a post-repair follow-up visit conducted 6–8 weeks post surgery. At this visit, fistula repair outcomes were determined and evaluation of any physical complications done. With financial (transport refunds and patient lodging support at the hospital) and logistics (surgical repair materials) support from an implementing partner, all enrolled women in this study returned for their post-surgical follow-up visit.

### Ethical approval

The RMH Ethics Committee provided ethical approval after the Study fulfilled the review of case records criteria (RMHSEC 002/03/14). No written/verbal consent was sought from the women with obstetric fistula as this study was based on a retrospective review of records at a time when study subjects could not be accessed.

### Data analysis

Case report data were manually transcribed from patient records into Microsoft Excel 2007 version (Microsoft Corp) by two independent data entrants. Data were then cleaned and exported into STATA version 12 (College Station, TX, USA) software for final analysis. Descriptive analysis was performed using means, ranges, standard deviations and proportions. Chi square tests were used to test for categorical differences in repair outcome across all socio- demographic and clinical characteristics. A multivariable logistic regression model was used to assess for determinants of repair outcome by including all variables that showed evidence of possible association with repair outcome (p < 0.25) in bivariate analysis. Results are presented as odds ratios with 95 % confidence intervals and the variable impact was considered significant at p < 0.05.

## Results

A total of 272 women with overall mean age of 34.6 years (SD ± 10.8) age range from 16 to 78 years were included. Most of fistula cases were aged >25 years (~83 %), were multigravida (78 %) and were having their index attempt at repair (86 %). Successful fistula repair outcome were achieved among 234 (86 %) and, among those with successful outcomes, 7 were found with stress incontinence at the follow-up visit conducted 6–8 weeks after surgery. The highest successful repair rates were achieved among who reported to have spent 0–2 days in labour (91 %) compared to those spent at least 3 days in labour (78 %). Two fistula cases were found with cervical cancer and deemed inoperable. The distribution of fistula cases by type was as follows, 93 (34.2 %) VVFs, 48 (17.6 %) RVFs, 65 (24 %) UVFs, 64 (23 %) VUFs with two women found with incontinence associated with bladder atony and a cystocele. A majority of ~70 % fistula were complications of obstructed labour while 26.5 % were caused by iatrogenic errors. Of the other 17 fistula, 6 were complications of infections, 7 were post-abortion complications, 2 were caused by trauma due to coitus and road traffic accident, while 2 women had fistula complications of cervical cancer. Details of age, obstetric characteristics and fistula repair outcomes are shown in Table 1.

**Table 1:** Baseline demographics, fistula characteristics and repair outcomes of 272 cases managed at a public tertiary hospital in Rwanda

Variable characteristics	Variable categories	N = 272	Repair outcomes	
		n (%)	Success (%)	Failure (%)
Age group	16–25	48 (17.3)	41 (85.4)	7 (14.6)
	≥26	224 (82.7)	193 (86.2)	31 (13.8)
Parity	1	60 (22.1)	50 (83.3)	10 (16.7)
	2–5	160 (58.8)	140 (87.5)	20 (12.5)
	≥6	52 (19.1)	44 (84.6)	8 (15.4)
Fistula type	VVF	93 (34.2)	79 (84.9)	14 (15.1)
	RVF	48 (17.6)	46 (95.8)	2 (4.2)
	UVF	65 (23.9)	51 (78.5)	14 (21.5)
	VUF	64 (23.5)	56 (87.5)	8 (12.5)
	Others	2 (0.8)	2 (100)	0 (0.0)
Fistula cause	Labour	186 (68.4)	159 (85.5)	27 (14.5)
	Surgical	71 (26.1)	64 (90.1)	7 (9.9)
	Others	15 (5.5)	10 (66.7)	5 (33.3)
Surgical operation	Vaginal	189 (69.5)	165 (87.3)	24 (22.7)
	Abdominal	62 (22.8)	53 (85.5)	9 (14.5)
	Others	19 (7.0)	16 (84.2)	3 (15.8)
	Inoperable	2 (0.7)	0 (0.0)	2 (100)
Time in labour (days)	0–2	177 (65.1)	161 (91.0)	16 (9.0)
	≥3	93 (34.9)	73 (78.5)	20 (21.5)
	Not applicable	2	0 (0.0)	2 (100)
Fistula duration (years)	0	99 (36.4)	92 (92.9)	7 (7.1)
	≥1	173 (63.6)	142 (82.1)	31 (17.9)
Repair attempt	1st	234 (86.0)	207 (88.5)	27 (11.5)
	2nd to 4th	38 (14.0)	27 (71.1)	11 (28.9)

### Predictors of successful outcomes after surgical intervention

By both univariate and multivariate logistic regression analysis, a history of having spent at least 3 days in labour (OR = 0.43, p value = 0.04), at least one previous failed attempt at repair [0.15 (95 % CI: 0.03–0.62), p value = 0.009] and having lived with the fistula for ≥1 year (OR = 0.26, p value = 0.013) were significant associated with poor repair outcomes (Table 2). Women whose fistula repair was done via the abdominal route had an 8.5 % (95 % CI: 0.005–1.571, p value = 0.078) lower odds of a successful repair compared to those with repair done through vaginal route, although this difference was borderline significant in our study. Interestingly, fistula type did not significantly influence repair outcomes in this study. The effects of predictor variables on repair outcomes are presented in Table 2.

**Table 2:** Univariate and multivariate analysis of determinants of successful fistula repair outcomes among 272 obstetric fistula cases

Case characteristics	Case characteristics	Unadjusted OR (95 % CI), P value^a^	Adjusted^b^ OR (95 % CI), P value^c^
Age group	16–25	1	–
	≥26	1.13 (0.46–2.76), 0.791	–
	1	1	–
	2–5	1.46 (0.64–3.36), 0.372	–
	≥6	1.224 (0.43–3.50), 0.705	–
Fistula type	VVF	1	–
	RVF	3.63 (0.78–16.96), 0.101	2.70 (0.52–14.05), 0.238
	UVF	0.53 (0.23–1.24), 0.144	0.58 (0.23–1.43), 0.234
	VUF	1.11 (0.42–2.88), 0.838	9.29 (0.46–187.26), 0.146
	Others	Empty	Empty
Fistula cause	Labour	1	–
	Surgical	1.39 (0.60–3.26), 0.440	–
	Others	0.67 (0.13–3.32), 0.621	–
Surgical operation	Vaginal	1	–
	Abdominal	0.69 (0.39–1.05), 0.292	0.183 (0.01–1.57), 0.078
	Others	0.81 (0.22–2.99), 0.752	0.43 (0.09–2.026), 0.288
	Inoperable	Empty	Empty
Time in labour in (days)	0–2	1	1
	≥3	0.35 (0.17–0.35), 0.005	0.43 (0.19–0.96), 0.040
	Not applicable	Empty	Empty
Fistula duration	0	1	–
	≥1	0.35 (0.15–0.84), 0.019	0.26 (0.09–0.75), 0.013
Repair attempt	1st	1	1
	2nd to 4th	0.34 (0.146–0.78), 0.011	0.15 (0.03–0.62), 0.009

^a^Chi square tests for binary characteristics were used to produce unadjusted p value

^b^Adjusted odds ratio were obtained after adjusting for variables; repair attempt, fistula duration in years, Time in labour, Surgical operation type and fistula type in final model

^c^Multivariate logistic regression tests were used to produce adjusted p value

## Discussion

In this retrospective review of medical records, a successful fistula repair rate among women undergoing their index closure attempt episode of 88.5 % was realised. Risk factor analysis identified parity, fistula type, underlying fistula cause, duration with fistula, type of surgical procedure, previous failed attempts to repair as variables that, alone or in unison, significantly affected surgical repair outcomes.

Quality obstetric care including availability of and attendance to ANC and presence of quality emergency obstetric surgeries are important factors in quality obstetric care and may serve to mitigate the burden of obstetric complications including development of obstetric fistula. ANC attendance—with the majority of first ANC visit occurring in second or third trimester, and health facility delivery rates of 98 and 69 % have been reported in Rwanda [19]. These high rates highlight the value in screening pregnant women to identify and actively follow-up those at risk of complicated pregnancies like teen mothers at risk of obstructed labour—implicated in causing 73.5 % of obstetric fistula in out study. However, provision of quality obstetric care is hampered by lack of medical personnel with the skills to conduct quality general surgical obstetric care at most health facilities and operationalization of strategies to prevent and/or effectively manage fistula complications in particular. Improving access to early and effective ANC services especially to women at community level is key to identifying potentially at-risk mothers and planning for optimal obstetric care. Although cause-of-fistula did not significantly influence fistula repair outcomes in this study, iatrogenic reasons accounted for 26.5 % of the fistula managed. The high proportion of iatrogenic caused fistula and the 14 % of women with prior failed fistula closures point to a need for improved obstetric management skills and specifically, fistula repair expertise through better training and mentorship. The almost 70 % obstetric cases related to obstructed labour point to a need for early identification, during ANC and in labour, of the most at risk mothers and scale up and increase in women attendance to ANC services.

In this study, about 86 % of all obstetric fistula repaired via the abdominal route had a successful outcome. Use of the abdominal route for repair was associated with borderline significant 0.18-fold lower odds of having a successful repair outcome compared to vaginally repaired fistula. Previous studies have shown mixed results with a study by Kriplani et al. showing significantly less incontinence (7.1 %) among women with obstetric fistula repaired vaginally compared to who were managed either via the abdominal approach or using a combined approach [21]. Nardos et al. compared repair outcomes between vaginal versus abdominal accessed fistula and, despite there being no significant difference in outcomes in the two arms, all of the failures in the vaginally repaired fistula group where to women with difficult to access fistula who were subsequently repaired abdominally with success outcomes achieved [22]. Other determinants of fistula closure outcomes including fistula access, severity of fistula scarring, skill of surgical repair and post-surgical care may also influence repair outcomes. In our study, route of access in fistula repair may have been cofounded by severity of damage of fistula with the abdominal route preferred for cases with severe, more difficult to access and often more scarred fistula.

In this study, overall fistula closure was achieved in 86 % while among those having their first fistula repair experience; a slight higher proportion of 88.5 % has a good repair outcome. These successful rates of closure are higher that WHO targets of >85 % as a measure of good quality of care [23]. In previous studies conducted in comparable settings, mixed findings with lower closure rates of 78 % in a Ugandan study and 73 % in a Zambian [24, 25] while in another study in Uganda, higher rates of >90 % were reported [26]. These differences in rate of successful repair could be attributed to difference in severity of cases handled per health facility, rationale for selection of type of repair procedure, skill of surgeons as well as differences in quality of fistula repair service offered at the difference study sites. These observations highlight the need to consider all determinants of successful repair outcomes particularly among women due for their index attempt at repair.

Delays in seeking health care and in reaching a health facility, which are key determinants in comprehensive emergency obstetric care, have been associated with poor health outcomes [27, 28]. In this study, about 35 % women reported having been in labour for ≥3 days while a majority of 64 % reported having lived with the fistula for ≥1 year. These findings highlight a major gap in limited availability of and access to comprehensive emergency obstetric care that is essential to mitigate the risks of obstetric emergences.

The leading cause of fistula in the developing world is obstructed labour. Unfortunately, limited quality obstetric care and other poorly characterized socio-economic factors may all lead to delay in seeking this care when it is available. Compared to being in labour for 0–2 days, women who reported having been in labour for >2 days had 2.5 higher odds of having a poor repair outcome in this study. The tissue damage and associated scarring due to ischemic necrosis of the soft tissues around the vagina and bladder and or rectum become worse the longer the duration of labour [16, 29]. Additionally, with limited surgical facilities, it is plausible that most obstructed labours management may involve more aggressive procedures such as instrument deliveries. With the majority of affected women living in resource-limited settings, the associated poor peri-operative care including lack of catheterizations further increases the risk of fistula development. In Rwanda hitherto, only four public hospitals currently offer fistula repair care highlighting the urgent need for increased access to these services [30].

About 64 % (173) women with obstetric fistula reported having had leakage from fistula for at least 1 year. Women who had lived with the fistula for greater 1 year had poorer odds for successful outcomes (1–2 years OR 0.24,  p value = 0.019 and 3–5 OR 0.41,  p value = 0.038). Among 581 women repaired in Kenya, Tanzania and Uganda, repairs done within 3 months of fistula development were more likely to achieve fistula closure (93.9 vs. 87.0 %) compared to repairs done after 3 months, respectively [31]. The longer the leakage continues, the more the obstetric fistula tissues becomes fibrotic and the fistula less amenable to surgical repairs. Delays in seeking may be partly due to limitations in knowledge of and access to available fistula repair services. Community-level initiatives to identify and support women with fistula towards increased aces to fistula repairs services are need to supplement vertically provided health facility based fistula repair services.

In this study, 13.8 % of the fistula repairs were conducted on women with a prior failed repeat attempts. Compared to first time repair attempts, women with at least one previous failed attends had a marginally significant reduced odds of success (OR 0.29, p value = 0.049) as reported elsewhere [22, 24, 32]. In one study, women who had a repeat fistula repair procedure were more likely to experience residual incontinence than those undergoing primary repair (20 vs. 10 %, p value = 0.006). However, this effect did not remain statistically significant in the multivariate analysis as reported elsewhere [33]. It is plausible that each additional fistula repair attempt leads to additional tissues damage and scaring and therefore, multiple repair attempts would reduce the chance of restorative physiological function. This highlights the need for skilful surgeons and the value of optimizing good fistula repair outcomes at the first fistula repair attempt.

### Implications for practice

Fistula closure rates higher that the WHO requirement for acceptable quality of care were realized at this facility demonstrating the feasibility of running a successful fistula repair services if the required infrastructure and human capacity resources are available. At least one in four fistula cases were causally attributed to iatrogenic factors in this study. This highlights the urgent need for training and mentorship to impart the required surgical skills to minimize risks of obstetric related surgical complications. While fistula repair care services need to be scaled up, supplementary strategies to support affected women with fistula in their communities to access fistula repair services may contribute to further mitigation of fistula burden.

### Study limitation

These study findings involving 272 women of difference ages, types and duration of fistula attending a public referral health facility in a developing country provides evidence of feasibility of achieving high successful fistula repair rates if infrastructural, medical expertise and patient support factors are favourable. Being a retrospective review of data collected as part of patient care records, key variable data associated with obstetric fistula repair outcomes that was unavailable include; degree of fistula scarring [15], fistula size [31], age at which fistula first developed [32, 34], bladder size [20], degree of damage in associated tissues, how repair procedures conducted were chosen, skill of surgeon and type of post-operative care. Absence of these data may have limited a more robust assessment of repair outcomes. In addition, health systems related factors like type of study participant selected, limited sample size, lack of recommended surgical repair materials, sub-optimal post operative care and non-standardized assessments or surgical procedures conducted may have influenced surgical outcomes.

## Conclusions

In this study, a fistula repair success rate of 86 % was recorded. Notably, histories of labour duration of ≥3 days, a prior failed attempt at fistula repair, and duration with fistula of ≥1 year were significant associated poor fistula repair outcomes. Given the high proportions of women with obstetric fistula caused by iatrogenic errors (26.5 %), prior failed repair attempts (14 %) and a duration with fistula of >1 year (63.6 %), mitigating fistula burden in Rwanda will require improvements in comprehensive emergency obstetric care, including scale up of services to increase access to fistula care, training of more fistula surgeons, early identification and appropriate referral of mothers at risk of obstructed labour as well as community-level identification of and provision of care to fistula affected women.
